# Recent updates on the role of microRNAs in prostate cancer

**DOI:** 10.1186/1756-8722-5-9

**Published:** 2012-03-14

**Authors:** Oudai Hassan, Aamir Ahmad, Seema Sethi, Fazlul H Sarkar

**Affiliations:** 1 Department of Pathology, Karmanos Cancer Institute, Wayne State University School of Medicine, Detroit, MI, USA; 2 Department of Oncology, Karmanos Cancer Institute, Wayne State University School of Medicine, Detroit, MI, USA

**Keywords:** miRNAs, Prostate Cancer, Carcinogenesis, Metastasis

## Abstract

MicroRNAs (miRNAs) are short non-coding RNAs that are involved in several important biological processes through regulation of genes post-transcriptionally. Carcinogenesis is one of the key biological processes where miRNAs play important role in the regulation of genes. The miRNAs elicit their effects by binding to the 3' untranslated region (3'UTR) of their target mRNAs, leading to the inhibition of translation or the degradation of the mRNA, depending on the degree of complementary base pairing. To-date more than 1,000 miRNAs are postulated to exist, although the field is moving rapidly. Currently, miRNAs are becoming the center of interest in a number of research areas, particularly in oncology, as documented by exponential growth in publications in the last decade. These studies have shown that miRNAs are deregulated in a wide variety of human cancers. Thus, it is reasonable to ask the question whether further understanding on the role of miRNAs could be useful for diagnosis, prognosis and predicting therapeutic response for prostate cancer (PCa). Therefore, in this review article, we will discuss the potential roles of different miRNAs in PCa in order to provide up-to-date information, which is expected to stimulate further research in the field for realizing the benefit of miRNA-targeted therapeutic approach for the treatment of metastatic castrate resistant prostate cancer (mCRPC) in the near future because there is no curative treatment for mCRPC at the moment.

## Introduction

Prostate cancer (PCa) is considered to be the most diagnosed cancer [1] and the second leading cause of cancer death in men older than 40 years of age in the USA [2]. The major problem of PCa is the development and acquisition of castrate resistant prostate cancer (CRPC) phenotype which eventually leads to the development of skeletal metastasis (mCRPC), at which point it becomes an incurable disease [1]. Therefore, investigations are underway to find the molecular basis of mCRPC so that novel therapeutic strategies could be devised. To that end many novel molecules are being tested and interrogated, among which microRNAs (miRNAs) are becoming an attractive area of research.

The miRNAs are small, noncoding subset of RNAs which consist of about 18-22 nucleotides and bind to the 3' untranslated region of messenger RNAs (mRNAs) [3]. By this action, they cause post-transcriptional inhibition or degradation of target mRNA, depending on the degree of complementary base pairing [4-6]. The miRNAs were first discovered in 1993 while studying *Caenorhabditis elegans *[7]. The first miRNA discovered was lin-4. It is a small, non-coding RNA molecule that was found to play a role in the development through a negative effect on lin-14 expression [7-9]. Seven years later, in 2000, let-7, the second miRNA was discovered, again in the *C. elegans *[9,10]. During the past 12 years, significant advances have been made in miRNA research leading to the discovery of over 4,500 miRNAs in vertebrates, flies, worms, plants, and viruses [9,11,12] out of which more than 1,000 miRNAs have been fully characterized and the number is expected to grow in the coming years. The miRNAs are being implicated in the regulation of an increasing number of physiological processes. It is also believed now that they play an important role in the regulation of many cellular functions ranging from maintenance to differentiation and tissue development, from metabolism to cell cycle [13-16]. All of these facts leads to the conclusion that aberrant expression of miRNAs will have impact on various biological processes where they are implicated, which will result in a variety of pathological events such as infection [13,17,18], cardiovascular diseases [19], neurodegenerative diseases [20] and, most importantly, cancer [13].

The role of miRNAs in cellular growth, differentiation and apoptosis of cancer cells through their interactions with their target mRNA has been studied [21-23]. miRNAs may be oncogenic or tumor suppressors [23,24], with oncogenic being up-regulated and the tumor suppressors being down-regulated in cancers. Generally, the importance of miRNAs in cancer is emphasized by the fact that around 50% of all miRNA genes are positioned in the so called 'fragile sites', the cancer associated genomic regions which are repeatedly changed in cancer. A lot of information has already been identified about aberrant miRNAs expression in cancers; the understanding of the functional importance of these aberrations has not been molecularly exploited [25].

The role of miRNAs in PCa is becoming clearer by understanding the interactions between miRNAs and their targets and the resulting impact on carcinogenesis of the prostate [23,26,27]. It is believed that several miRNAs and their targets are aberrantly expressed in PCa which, in turn, alter the cellular growth, invasion, and metastatic potential of prostate cancer cells. The abnormal expressions of certain miRNAs are now considered valuable biomarkers for diagnosis, prognosis and classification of PCa [23,28,29]. All of the above information underscores the importance of the biology of miRNAs in PCa. Their specific abnormalities, and how one could regulate their expressions will likely become novel avenues by which newer therapeutic strategies could be developed for the treatment of mCRPC.

### miRNA biogenesis

The biogenesis of miRNA involves many critical steps; the initial phase is the transcription by RNA polymerase II that leads to the formation of primary miRNA (pri-miRNA) which comprises of hundreds to thousands of nucleotides [9,30]. The second phase is catalyzed by a ribonuclease called ribonuclease III (RNase III), Drosha. This step leads to the split of pri-miRNA and results in what is called a precursor miRNA (pre-miRNA), which usually comprises of around 70 nucleotides, and this phase is accomplished with the help of dsRBD protein, DGCR8, a protein that helps to ensure a perfect and efficient processing of pri-miRNA into pre-miRNA [9,31,32]. Thereafter, a nuclear export factor, called exportin 5, binds to the pre-miRNA and transports it into the cytoplasm where the next processing phase take place [9,33,34]. Here another RNase III, dicer, interacts with the pre-miRNA, and the outcome of this process is the formation of a RNA duplex of around 22 nucleotides, which is the mature miRNA consisting of double-stranded duplex. Dicer usually operates with the help of a different dsRBD protein, the trans-activator RNA (tar)-binding protein (TRBP) [9,35,36]. Subsequently, all these steps allow the mature miRNA cooperate with the RNA-induced silencing complex (RISC) [9,37,38], and ultimately allow miRNA to control post transcriptional regulation of functional mRNAs.

### miRNA and cancer stem cells in prostate cancer

Cancer stem cells (CSCs), also called tumor-initiating cells, are a group of cells which play an important role in the progression of cancer and its metastasis [39]. The CSCs hypothesis assumes that cancers are basically derived from a small fraction of cancer cells that have exclusive ability to self-renew and initiate/maintain the tumor [40,41]. This specific ability of CSCs allows to initiate the development of cancer as documented first in human leukemia [42,43]. For the isolation and identification of CSCs many markers have been used. Early on, CD44 (an adhesion molecule) was used to identify CSCs, individually or sometimes in combination with other markers. It was shown that Prostate CSCs that have increased clonogenic potential [44], and tumor initiating ability and metastasis [45,46] capacities, have increased CD44^+ ^cell population.

Emerging evidence suggests that miRNAs may function as the regulators of CSC characteristics, as documented by many studies [47-50]. Studies have clearly shown that the expression levels of certain miRNAs in stem cells are different from other normal tissues [40,51], suggesting that miRNAs are important regulator of CSC function. One example is miR-34a, a p53 target [52-56], which has strong anti-tumor and anti-metastasis effects [57]. Studies have shown that miR-34a is down regulated in CD44^+ ^PCa cells, and its forced expression in CD44^+ ^PCa cells resulted in the inhibition of clonogenic growth and inhibition of metastatic behavior and tumor regeneration [57]. The miR-34a was also established as an important negative regulator of CD44^+ ^PCa cells, and it is assumed that this decreased expression of miR-34a in CD44^+ ^PCa cells (including CSCs) plays an important role in PCa development and metastasis [57]. Moreover, the expression of miR-34a antagomirs in CD44^- ^prostate cancer cells promoted tumor development and metastasis [57].

Taking into consideration the prevalent expression of CD44 in CSCs and the role of CD44 in mediating CSC migration and homing, suggesting its role in metastasis in various malignancies including PCa whereby the role of miR-34a in controlling CSC characteristics appears to be important, and thus targeting of miR-34a pathways could become innovative treatment strategies for PCa [57]. Further studies are underway in order to establish the molecular interplay between miRNAs and CSCs in PCa and other human malignancies.

### The role of miRNAs in the processes of Epithelial-to-Mesenchymal Transition (EMT) in PCa

The process of EMT is defined as one when epithelial cells acquire phenotypic characteristics of mesenchymal cells whereby the epithelial cells change their epithelial cobblestone phenotype to a mesenchymal elongated fibroblastic phenotype, which contributes to increased cell motility and invasion that is required for metastatic process. During EMT, cancer cells lose epithelial cell-cell junctions associated with a decrease in the expression of epithelial proteins such as E-cadherin and junction plakoglobin, and the increase in expression of mesenchymal markers such as vimentin, fibronectin and α-smooth muscle actin. These changes are also associated with augmented activity of matrix metalloproteinases (MMPs), such as MMP2, MMP3, MMP9, which leads to an invasive phenotype [58]. All of the above mentioned processes lead to increased invasion and migration in many cancers including PCa [59]. Because the miRNAs are part of the cellular signaling circuit that controls EMT [60], it has been suggested that many miRNAs families, including miR-200 family and miR-205, play important roles in controlling EMT [61-63].

The miRNA 143 and miRNA 145 are other two miRNAs that are assumed to play a role in EMT. In PCa, miR-143 and miR-145 are deregulated in primary cancer compared with normal prostate tissue [64-67]. The up-regulation of miR-143 in prostate cancer cells represses mesenchymal markers (vimentin and fibronectin) and increases the epithelial marker E-cadherin [1], while the up-regulation of miR-145 leads to the same effects except for vimentin [1]. The previous information shows that miRs-143 and -145 may be suppressors of EMT, and based on the fact that EMT plays an important role in invasion and migration consistent with mesenchymal characteristics allows detachment and movement of cells from the primary tumor [68]. Therefore, it can be assumed that miRs-143 and -145 inhibit invasion and migration in prostate cancer.

The role of most miRNAs in regulating EMT is still not a clear-cut with just a handful of miRNAs being evaluated for their role in EMT of PCa [1,69,70] (Table 1). With the crucial role of miRNAs in EMT, it is expected that the regulation of EMT by miRNAs in PCa will be the center of many cutting-edge research in the upcoming years.

**Table 1:** Regulation of EMT in PCa by miRNAs

miRNA	Effect in PCA	Reference
miR-200b	Down-regulation of ZEB1, ZEB2 and Snail 2	Kong 2009 [69]

miR-200 family	Reverses EMT and down-regulates Notch-1 and Lin28B	Kong 2010 [70]

miR-143 and miR-145	Suppression of mesenchymal markers and up-regulation of epithelial marker	Peng 2011 [1]

Let-7 family	Expression of epithelial markers	Kong 2010 [70]

### The relationship between circulating miRNAs and tumor progression in prostate cancer

Circulating miRNAs have been suggested as encouraging biomarkers for noninvasive diagnosis in many tumors [71]. It has been proposed that miRNAs profiles in tumor cells have a prognostic value for some cancer patients, and a similar correlation with serum miRNAs profiles should be a viable approach [71].

Brase et al. [71] found that miR-375 was the top marker in a screening study (metastatic vs. localized prostate cancer) and that its expression is higher in prostate cancer tissue compared to normal epithelium. They also found that it was considerably related to lymph-node status of the prostate cancer patients, but no significant difference was observed in the serum levels of patients with gleason score 8 tumors and gleason score 7 tumors. So, it seems that the circulating level of miR-375 is associated mainly with systemic disease (lymph node involvement and metastasis) rather than the grading of primary prostate cancer [71]. However, the definite role of miR-375 in prostate cancer is still not very clear.

The role of miR-141 and miR-200b (both belong to the same family of miRNAs) were reported to be the two largely over regulated miRNAs in prostate epithelial cells in comparison with prostate stromal cells [71]. But the circulating levels of miR-141 were found to be much higher in the serum of patients with high-risk tumors when compared to intermediate-risk samples. Also, circulating levels of miR-141 have been found to differentiate between patients with metastatic PCa and healthy controls [71].

Based on these limited studies, one could conclude that circulating miRNAs may offer good perspective as noninvasive biomarkers for tumor progression, including prostate cancer, but further research has to be done in this field in order reach a better understanding of the role of miRNAs in the serum or plasma [71].

### The specific roles of certain microRNAs in prostate cancer

As mentioned above, miRNAs are known to play important roles in the progression of different cancers, including PCa. Some miRNAs can function as tumor suppressors wherein their elevated levels are indicators of good prognosis. On the contrary, other miRNAs are promoters of carcinogenesis and their expression levels are elevated in advanced stage of some cancers, which clearly suggests that these miRNAs may offer attractive targets for therapy. In this section, we will discuss the roles of certain miRNAs in prostate cancer as summarized in Table 2.

**Table 2:** miRNAs that influence PCa progression

miRNA	Role in PCa	Function	Study
miR-15a and miR-16	Tumor suppressors	Inhibit cell proliferation, invasion and angiogenesis through regulation of multiple targets	Aqeilan 2010 [25], Musumeci 2011[72]

miR-21	Onco-miRNA	Increases tumor growth, invasion and metastasis	Si 2007 [79], Selciklu 2009 [80], Li 2009 [81], Ribas 2009 [82]

miR-125b	Onco-miRNA	Increases cell proliferation and inhibits apoptosis	Lee 2005 [84], Shi 2007 [26], Vere White 2009 [85]

miR-143	Tumor suppressor	Inhibits cell proliferation and migration by regulating KRAS, MAPK pathways and cell cycle. Also inhibits metastasis	Clape 2009 [67], Xu 2011 [4], Friedman 2009 [73]

miR-145	Tumor suppressor	Inhibits migration, invasion and metastasis	Friedman 2009 [73]

miR-200 s	Tumor suppressor	Inhibit cell migration and invasion by reversing EMT	Kong 2009 and 2010 [69,70]

miR-221	Onco-miRNA	Stimulates cell growth and influences cell cycle progression	Zheng 2011 [78], Galardi 2007 [77], Sun 2009 [76], Pang 2010 [23].

miR-222	Onco-miRNA	Increased cell cycle progression	Galardi 2007 [77], Sun 2009 [76], Pang 2010 [23].

miR-488	Tumor suppressor	Inhibits Androgen Receptor-mediated cell growth	Sikand 2010 [75]

### Tumor suppressor miRNAs

#### The role of miRNA-15a and miRNA-16

Both of these miRNAs work as tumor suppressors mediated through deregulation of multiple oncogenes, and these oncogenes include: BCL2, MCL1, CCND1, and WNT3A [25]. The aforementioned oncogenes can promote cell proliferation, survival and invasion [25]. The down regulation of these miRNAs has been reported in many malignancies including: CLL, Pituitary adenoma, and Prostate carcinoma [25]. The miR-15a and miR-16 are both located at 13q14.3, and the deletions at this location have been reported in many malignancies including: CLL, MM, Mantle cell lymphoma, and Prostate carcinoma [25]. In a recent study, the expression of miR-15a, miR-16-1 in PCa samples showed consistent down regulation of these genes in around 80% of cancer samples compared with that of normal samples [25]. Studies have also shown that miR-15a, miR-16-1 are down regulated in pituitary adenomas in comparison with normal pituitary, which basically enhances the assumption that they work as tumor suppressors and that their knock down by allelic loss may contribute to tumorigenesis.

BCL2 is an oncoprotein that performs an important role in the genetic program of the eukaryotic cells, it prevents cell death and its over-expression was found to be related to many cancers such as: leukemia, lymphoma, and carcinomas in general [25]. It was reported that miR-15a, miR-16-1 sequences and BCL2 mRNA sequences share a complementary homology, and thus the previous information collectively suggests that miR-15a, miR-16-1 could suppress BCL2 by post transcriptional repression [25]. It has been reported that miR-15a, miR-16-1 cluster targets not only BCL2 but also CCD1 (encoding cyclin D1) and WNT3A mRNAs, which promote many prostate carcinogenic features including; survival, proliferation, and invasion [25]. The *in vivo *knock down of miR-15a, miR-16-1 resulted in hyperplasia associated with CCD1 and WNT3A up regulation, all of the above evidence suggest that loss of miR-15a and miR-16-1 may be a significant pathogenic event during the development of PCa [25].

The miR-15 and miR-16 are usually down-modulated in the tumor sustaining stroma, an observation that can be explained by the effect of cancer cells on the stroma [72]. The miR-15 and miR-16 have a tumor suppressor activity on both cancer cell level and at the stromal microenvironment [72]. Lately, it was also proposed that miR-15 and miR-16 direct the expression of VEGF and IL-6, two factors that stimulate tumor angiogenesis and bone metastasis, respectively. Moreover, it was shown that re-expression of miR-15 and miR-16 in cancer-associated fibroblasts (CAFs) will cause attenuation of the stromal support capability, and this will result in the decrease in cell proliferation and migration in primary and metastatic tumors [72]. These observations lead us to conclude that in the context of prostate cancer, miR-15 and miR-16 are tumor suppressors, at least, on two levels such as at the levels of tumor cell and stromal cells.

### The role of miR-143 and miR-145

We have discussed above the role of miR-143 in EMT. Studies have shown that miR-143 is considerably decreased in PCa, and its expression is further decreased during cancer progression [67]. K-RAS (V-Ki-ras2 Kirsten rat sarcoma), a key molecule of EGFR/RAS/mitogen-activated protein kinase (MAPK) signaling pathway, is a viral oncogene homolog that was incriminated in cell proliferation and migration in response to growth factors. The MAPK pathway also works at another level through its effect on Androgen receptors (AR), where it increases AR in response to low androgen, and this is considered a main process in Androgen derivation therapy relapse. K-RAS is a potential target of miR-143 [73], thereby lower levels of miR-143 in prostate cancer cells may be incriminated in carcinogenesis due to the lack of its inhibitory effect on K-RAS and MAPK pathway.

Xu et al. [4] showed that miR-143 regulates K-RAS, p-ERK1/2, and cyclin D1 and plays a role in cell proliferation, migration, and chemosensitivity in prostate cancer. They also showed that miR-143 over-expression in prostate cancer cells represses proliferation and migration, thereby augmenting sensitivity to docetaxel by affecting EGFR/RAS/MAPK pathway. The expressions of miRNAs-143 and -145 (another miRNA which is assumed to be tumor suppressor) were found to be down-regulated considerably in metastasis samples [73]. Exploring the correlation of the levels of miRNAs-143 and -145 with clinico-pathological features of PCa showed that down-regulation of miRNAs-143 and -145 were negatively associated with bone metastasis, the gleason score and the levels of free PSA in primary PCa patients [73]. Over-expression of miR-143 and -145 by retrovirus transfection decreased the ability of migration and invasion *in vitro*, and tumor development and bone invasion *in vivo *of PC-3 cells (a human PCa cell line originated from a bone metastatic PCa specimen) [73]. Their up-regulation also enhanced E-cadherin expression and decreased fibronectin expression in PC-3 cells with features of a less invasive morphologic pattern [73].

The information above suggests that miRs-143 and -145 are related to bone metastasis of PCa and may play a biological role in this process [73]. One could postulate that the possibility of using them in the clinical setting as biomarkers to individualize different stages of human PCa and could predict the development of bone metastasis in patients well ahead of time [73]. Even though up-regulation of miRNAs-143 and -145 are found to suppress the aggressiveness and EMT of PC-3 cells with regard to bone metastasis, it did not have the same effects on LNCaP cells that was derived from lymph node metastasis [73]. Actually, deregulation of miRs-143 and -145 was not found in lymph node metastasis in comparison to primary PCa specimens. This information suggests that miR-143 and -145 may have a cell type-specific activity and could suppress only bone metastasis without inhibiting lymph node metastasis, and that the loss of these miRNAs could selectively promote metastasis, which could be due to deregulated expression of other miRNAs such as miR-221 [73].

### The role of miRNA-200

Studies from our laboratory have shown that the miR-200 family controls epithelial-mesenchymal transition (EMT) by targeting zinc-finger E-box binding homebox 1 (ZEB1) and ZEB2 [63,69,74]. There is enough evidence to suggest that the processes of EMT can be elicited by various growth factors, such as transforming growth factor β and platelet-derived growth factor-D (PDGF-D), which is expressed in PCa tissue [69], and it was shown that over-expression of PDGF-D in PC3 cells (prostate cancer cell lines with high metastatic potential) leads to the acquisition of the EMT phenotype [69]. It was also proved that significant down-regulation of the miR-200 family in PC3 PDGF-D cells and in PC3 cells exposed to purified active PDGF-D protein, resulting in the up-regulation of ZEB1, ZEB2, and Snail2 expression (a transcription factor which belongs to the snail protein family and plays critical roles in the formation of tissues during embryonic development) [69]. Interestingly, re-expression of miR-200b in PC3 PDGF-D cells led to reversal of the EMT phenotype accompanied with the down-regulation of ZEB1, ZEB2, and Snail2 expression, and all of these events were associated with greater expression levels of epithelial markers [69]. Moreover, it was proved that transfection of PC3 PDGF-D cells with miR-200b considerably decreased the expression of ZEB1, ZEB2, and Snail2 at both the mRNA and protein levels, with simultaneous greater expression levels of epithelial markers such as E-cadherin, stratifin, CRB3, EpCAM, F11R, and connexin 26, all of these events collectively led to the inhibition of cell migration and invasion [69]. In a breast cancer model, our studies have shown that *in vivo *manipulation of miR-200b leads to significantly reduced pulmonary metastases of breast cancer cells [74] which further supports the role of miR-200 family in metastases of human cancers.

It is well known that NF-κB plays an essential role in facilitating the processes of EMT induced by different factors through up-regulation of ZEB1 and ZEB2, which in turn suppress the expression of miR-200 family members by binding to the E-box sequence of the miR-200 promoter [69]. Whereas miR-200 can down-regulate the expression of ZEB1 and ZEB2 by interacting with the 3'-UTR of ZEB1 and ZEB2 mRNA [69]. All of these findings suggest a double-negative feedback loop between miR-200 and ZEB1/ZEB2 that permits the preservation of the EMT phenotype, even after withdrawal of the initial inducing signal, which might become a critical target for the reversal of EMT [63]. From these facts, we can conclude that PDGF-D-stimulated attainment of the EMT phenotype in PC3 cells is, partly, an outcome of suppression of miR-200 and that new strategies in which miR-200 would be up-regulated will become an auspicious approach for the treatment of invasive prostate cancer [69].

### The importance of miRNA-488

Another miRNA that has been associated with PCa is miR-488. The miR-488 is encoded through AsTN1 gene. Two mature molecules results from the processing of its precursors: miR-488* and miR-488 and both of them are expressed predominantly in human brain tissue [75]. Studies have shown that miR-488* was not expressed in several prostate cancer cell lines, although the etiology is still unclear and thus it is currently being further investigated [75].

Androgen receptor is a direct target of miR-488, as miR-488 has a binding site at the 3'UTR of AR gene where it binds and suppresses its expression [75]. It was shown that cells transfected with miR-488 results in reduced expression of AR in both Androgen-dependent (LNCaP) and Androgen-independent (C4-2B) PCa cells. In both the cell lines, treatment with miR-488 mimics was found to retard the growth of these cells, but this was not the fact with AR-negative DU145 cells [75], suggesting the regulatory role of miR-488 on AR expression. These results suggest that miR-488 could function as a tumor growth suppressor, which is mediated by deregulation of AR expression [75].

Although it is still too premature to make conclusions, the results of these studies clearly showed that miR-488* transfection into LNCaP and C4-2B cells led to the repression of AR expression, thereby suggesting that finding a way to increase the levels of endogenous miR-488* could have a great impact on designing novel treatment strategies for PCa.

### Tumor promoter oncomiRNAs

The miRNA expression profiling analyses have shown that many miRNAs are up-regulated in prostate cancer [23]. These oncogenic miRNAs suppresses the apoptosis-related genes, so their over-expression leads to increased tumor growth and metastasis [23].

### The role of miRNA -221 and miRNA-222

The miR-221 and miR-222 are both considered as oncogenic and were found to be associated with the development and metastasis of prostate cancer [23]. One of the methods through which these miRNAs elicit their effect is by binding to one of their target mRNA, p27kip1 and cause suppression, which results in tumor growth [76,77]. Another action for them is their role in the development or maintenance of castration-resistant prostate cancer (CRPC), phenotype through a mechanism that is not yet clearly understood, although it may be through influencing response of AR-mediated signaling in prostate cancer cells [76].

With regards to miR-221, studies have shown that miR-221 levels are up-regulated in both ADPC and AIPC compared to normal control [78]. One pathologic process that plays an important role in the carcinogenesis and hormone therapy failure in PCa is neuroendocrine differentiation (NE), a process that is associated with tumor progression and poor prognosis. Studies have shown that miR-221 is capable of inducing NE differentiation in LNCaP cells in an androgen deprived environment, which may lead to Androgen Independence (AI) [78]. It was revealed that miR-221 stimulates the growth of LNCaP and LNCaP-AI cells, and it is consistent with findings that the ectopic introduction of miR-221 in low expressing LNCaP cells bolstered their growth potential by inducing a G1-S shift in cell cycle [78].

One paradox to mention about miR-221 is that although the expression of miR-221 is higher in LNCaP-AI cells (which are more invasive) compared to LNCaP (which are less invasive), suggesting that miR-221 promotes invasion of PCa cells [78] whereas the up-regulation of miR-221 in LNCaP cells did not increase its ability of increased cell migration, while the invasion capacity of LNCaP-AI cells was deregulated by knock-down of miR-221 expression. The aforementioned facts can be explained by assuming that other pathways are involved in regulating the migration capability of cells during the progression of ADPC into AIPC. It was further concluded that miR-221 could influence PCa via regulation of DVL2 (Dishevelled 2), which in summary is an important intracellular mediator of the WNT signaling pathway. An important target gene for WNT is MMP-7 (a member of cellular adhesion molecules which controls cellular adhesion, invasion, and migration), and the activation of MMP-7 was found to greatly strengthen the capability to destroy extracellular matrix, especially in cancer cells [78], suggesting that miR-221 may play important role in the regulation of invasion and metastasis.

### The role of miR-21

Another onco-miRNA is miR-21; it is usually up-regulated in prostate cancer and plays a role in tumor growth, invasion, and metastasis [79,80]. Lately, miR-21 was individualized as an oncogene which is up-regulated in various cancers (glioma, breast cancer, colorectal cancer, stomach/gastric cancer, hepatocellular carcinoma, pancreas cancer, lung cancer, cholangiocarcinoma, leukemic cancer, and prostate cancer etc.) [81]. Anti-sense studies of miRNA-21 in glioblastoma cell lines revealed that it directs cell growth by inhibiting apoptosis while it does not influence cell proliferation [81]. Recent studies have revealed an increase in apoptosis in DU145 and PC-3 cells after blocking miR-21 function, while LNCaP cells, which have low level of miR-21, showed no changes in apoptosis in response to miR-21 blockade [81]. The information mentioned above insinuated that miR-21 plays an important role in the resistance to apoptosis observed in DU145 and PC-3 cells [81]. These results also suggest that miR-21 might have a role to play in AR-negative cells but this needs to be further investigated.

Many genes have been identified as targets of miR-21 in carcinogenesis including; TPM1, PDCD4, and MARCKS [81]. It has been shown that MARCKS (a gene which encodes myristoylated alanine-rich C-kinase substrate) has high frequency frameshift mutations during carcinogenesis in hereditary nonpolyposis colorectal cancer (HNCC) [81]. It was also revealed that the expression of MARCKS is down-regulated in HCC tissues in comparison with cirrhotic, benign liver tissues [81]. MARCKS plays a part in TPA-mediated cellular migration in neuroblastoma, probably through its effect as a downstream target of Protein kinase C epsilon [81]. All these results suggest that MARCKS plays a role in tumorigenesis, and the effect of miR-21 on PCa cell motility and invasion may in part be due to its regulation of MARCKS gene. Recent studies have also shown that in the presence of androgen, AR can bind to miPPR-21, a miR-21 promoter, and this results in the over-expression of miR-21 at its transcription level, leading to castration resistance [82]. In support of what has been mentioned above, anti-miR-21 may augment the sensitivity of prostate cancer cells to apoptosis [81], and also negatively affect the motility and invasive characteristic of cancer cells [81].

In summary, it appears that miR-21 plays an essential role in apoptosis and metastasis of PCa [81]. Since no effective therapy is available to cure PCa, in part due to the resistance of androgen-independent advanced prostate cancer cells to apoptotic death, gene therapy that targets miR-21 may be a potential alternative therapy for androgen-independent PCa that have an up-regulated expression of miR-21. Moreover, it is tempting to speculate that natural agents (nutraceuticals) could serve as a therapeutic strategy for PCa because many of these agents could inhibit the expression of miR-21 as suggested by our recent results in pancreas cancer [83].

### The role of miRNA-125b

Another onco-miRNA is miR-125b which is considered very important for cell proliferation [84] and it is over-expressed in prostate cancer [23]. It has been reported that the reduction of miRNA-125b was found to be associated with the regulation of cellular proliferation of cancer cells, and this effect was attenuated by co-transfection of mature miRNA [84]. The biological role of miR-125b in PC-3 cells was studied and it was revealed that the depletion of miR-125b by numerous transfections of si-125b2 was followed by a substantial proliferation defect in PC-3 cells [84]. This growth defect was not associated with aberrant accumulation of cells in one stage of cell cycle or by apoptosis. Also, the depletion of miR-125b by 2'-O-methyl oligonucleotide showed the same phenotype with a similar effect on cell proliferation [84].

Studies have also shown that transfection of mature synthetic miR-125b causes PCa cell growth [84,85] and this was in part due to its effect on the 3'UTR of BAK1 (a pro-apoptotic member of the BCL-2 gene family that is involved in initiating apoptosis) transcript [26]. Nonetheless, down-regulation of BAK-1 only could not attenuate miR-125b's growth stimulatory effect, suggesting that there are other targets for miR-125b in prostate cancer cells [26,85]. Some recent reports individualized EIF4EBP1 (Eukaryotic translation initiation factor 4E-binding protein 1, a gene that encodes one member of a family of translation repressors proteins) as another specific target for miR-125b in PCa [65], but it is still not conclusive, suggesting that future in-depth investigation is warranted.

## Conclusions and perspectives

Research investigations focused on miRNAs have suggested a strong prognostic and therapeutic importance of miRNAs in PCa. Such studies have established an intimate relationship between prostate cancer and miRNA with emerging data clearly suggesting that miRNA is a very promising field although further in-depth mechanistic studies are required to ascertain the role of specific miRNA(s) and relevant target(s) in the development and progression of PCa especially the emergence of metastatic castrate resistant prostate cancer. Once we gain additional scientific knowledge it would be easier to focus on the development of strategies for up-regulation and down-regulation of specific miRNA as a novel and targeted therapeutic approach for the treatment of PCa. For example, our own studies with miR-21 in pancreatic and colon cancer models have shown that targeted regulation of this miRNA can lead to effective anticancer therapy [83,86-90]. As discussed above, miR-21 has been implicated in PCa as well and, therefore, our strategy may also work in PCa, which requires in-depth mechanistic pre-clinical studies. Together, we believe that identification of key miRNAs and their targets that are intimately involved with the development and progression of PCa and parallel development of strategies for deregulation of miRNAs would allow inhibition of tumor growth, invasion, angiogenesis and metastasis in PCa, which is expected to be clinically useful for the management of patients diagnosed with PCa.

## Competing interests

The authors declare that they have no competing interests.

## Authors' contributions

OH and AA surveyed the literature and drafted manuscript. SS participated in the design of this article. FHS conceived of the study and participated in its design, coordination and finalization of the manuscript. All authors read and approved the final manuscript.
